# Supplementary material for: Genomic insights into a versatile deep-sea methanotroph constituting the rare biosphere of a Brazilian carbonate mound complex
Source: mSystems. 2025 Dec 23;11(2):e01311-25. doi: 10.1128/msystems.01311-25 (PMC12911358; doi:10.1128/msystems.01311-25)
Supplement: Supplemental Material — Tables S1 to S5; Fig. S1 to S4. [file msystems.01311-25-s0001.docx]

**Genomic Insights into a Versatile Deep-Sea Methanotroph Constituting the Rare Biosphere of a Brazilian Carbonate Mound Complex**

Ana Carolina de Araújo Butarelli ^1*^, Fernanda Mancini Nakamura ^1^, Francielli Vilela Peres ^1^ Flúvio Modolon da Silva ^1^, Amanda Gonçalves Bendia ^1^ , Raissa Basti ^1^ , Michel Michaelovitch de Mahiques ^1^, Paulo Yukio Gomes Sumida ^1^,Vivian Helena Pellizari ^1^

^1^  Oceanographic Institute, University of São Paulo, 05508-120, São Paulo, Brazil

* Corresponding author: anacarolinabutarelli@usp.br

**Supplementary Table 1.** Sediment sample collection metadata. Description of the sediment samples collected using a box-corer, with a description of the collected area, season, type of sample and location.

| **Sampling area** | **ID Station** | **Sampling method** | **Sample Type** | **Latitude** | **Longitude** | **Depth (m)** |
| --- | --- | --- | --- | --- | --- | --- |
| Area 1 | *St.* 681 | Box-corer 1 | Sediment | 24˚ 31.422’ | 043˚ 55.825’ | 810 |
| Area 1 | *St.* 681 | Box-corer 2 | Sediment | 24˚ 31.399’ | 043˚ 55.872’ | 744 |
| Area 1 | *St.* 681 | Box-corer 3 | Sediment | 24˚ 31.328’ | 043˚ 55.849’ | 740 |
| Area 1 | *St.* 683 | Box-corer 1 | Sediment | 24˚ 37.452’ | 044˚ 00.878’ | 852 |
| Area 1 | *St.* 683 | Box-corer 2 | Sediment | 24˚ 37.449’ | 044˚ 00.883’ | 865 |
| Area 1 | *St.* 683 | Box-corer 3 | Sediment | 24˚ 37.449’ | 044˚ 00.885’ | 865 |
| Area 1 | *St.* 684 | Box-corer 1 | Sediment | 24˚ 40.281’ | 044˚ 04.943’ | 820 |
| Area 2 | *St.* 685 | Box-corer 1 | Sediment | 24˚ 55.946’ | 044˚ 28.419’ | 592 |
| Area 2 | *St.* 686 | Box-corer 1 | Sediment | 24˚ 55.200’ | 044˚ 35.483’ | 581 |
| Area 2 | *St.* 686 | Box-corer 2 | Sediment | 24˚ 55.228’ | 044˚ 35.406’ | 577 |
| Area 2 | *St.* 686 | Box-corer 3 | Sediment | 24˚ 55.247’ | 044˚ 35.415’ | 578 |
| Area 2 | *St.* 687 | Box-corer 1 | Sediment | 24˚ 54.012’ | 044˚ 28.467’ | 675 |
| Area 2 | *St.* 687 | Box-corer 2 | Sediment | 24˚ 53.997’ | 044˚ 28.536’ | 665 |
| Area 2 | *St.* 687 | Box-corer 3 | Sediment | 24˚ 54.065’ | 044˚ 28.506’ | 677 |
| Area 2 | *St.* 688 | Box-corer 1 | Sediment | 24˚ 53.536’ | 044˚ 32.181’ | 564 |
| Area 2 | *St.* 688 | Box-corer 2 | Sediment | 24˚ 53.534’ | 044˚ 32.141’ | 563 |
| Area 2 | *St.* 688 | Box-corer 3 | Sediment | 24˚ 53.517’ | 044˚ 32.122’ | 563 |
| Area 3 | *St.* 689 | Box-corer 1 | Sediment | 26˚ 15.742’ | 045˚ 42.869’ | 869 |
| Area 3 | *St.* 689 | Box-corer 2 | Sediment | 26˚15.’770' | 045˚ 42.858’ | 870 |
| Area 3 | *St.* 690 | Box-corer 1 | Sediment | 26˚ 11.560’ | 045˚ 38.728’ | 762 |
| Area 3 | *St.* 690 | Box-corer 2 | Sediment | 26˚ 11.553’ | 045˚ 38.728’ | 739 |
| Area 3 | *St.* 690 | Box-corer 3 | Sediment | 26˚ 11.560’ | 045˚ 38.723’ | 736 |
| Area 3 | *St.* 691 | Box-corer 1 | Sediment | 26˚ 53.216’ | 046˚ 24.732’ | 519 |
| Area 3 | *St.* 691 | Box-corer 2 | Sediment | 26˚ 53.219’ | 046˚ 24.730’ | 516 |

**Supplementary Table 2.** Reference genomes obtained from the National Center for Biotechnology Information (NCBI) for comparative analysis with Metagenome-Assembled Genomes (MAGs). The table lists accession numbers, taxonomic designations at the species/strain level, and source environments of *Methylotuvimicrobium* genomes. Included references span diverse habitats: *Methylotuvimicrobium alcaliphilum 20Z* (pure culture), *Methylotuvimicrobium buryatense SGB1C* (soda lake sediment), and *Methylotuvimicrobium* sp. wino1 (marine sediment enrichment culture).

| **Access Number** | **Reference Genome** | **Environment** |
| --- | --- | --- |
| PRJEA73721 | *Methylotuvimicrobium alcaliphilum* 20Z | Pure culture |
| PRJNA515283 | *Methylotuvimicrobium buryatense* 5GB1C | Soda lake sediment |
| NZ_CP024202.1 | *Methylotuvimicrobium* sp. wino1 | Enrichment culture inoculated with marine sediment |

**Supplementary Figure 1.** Venn diagram of microbial cultures. The diagram shows the distribution of microbial taxa identified under aerobic and anaerobic conditions. A total of 133 ASVs were unique to aerobic conditions, 112 were unique to anaerobic conditions, and 133 taxa were shared between both environments.


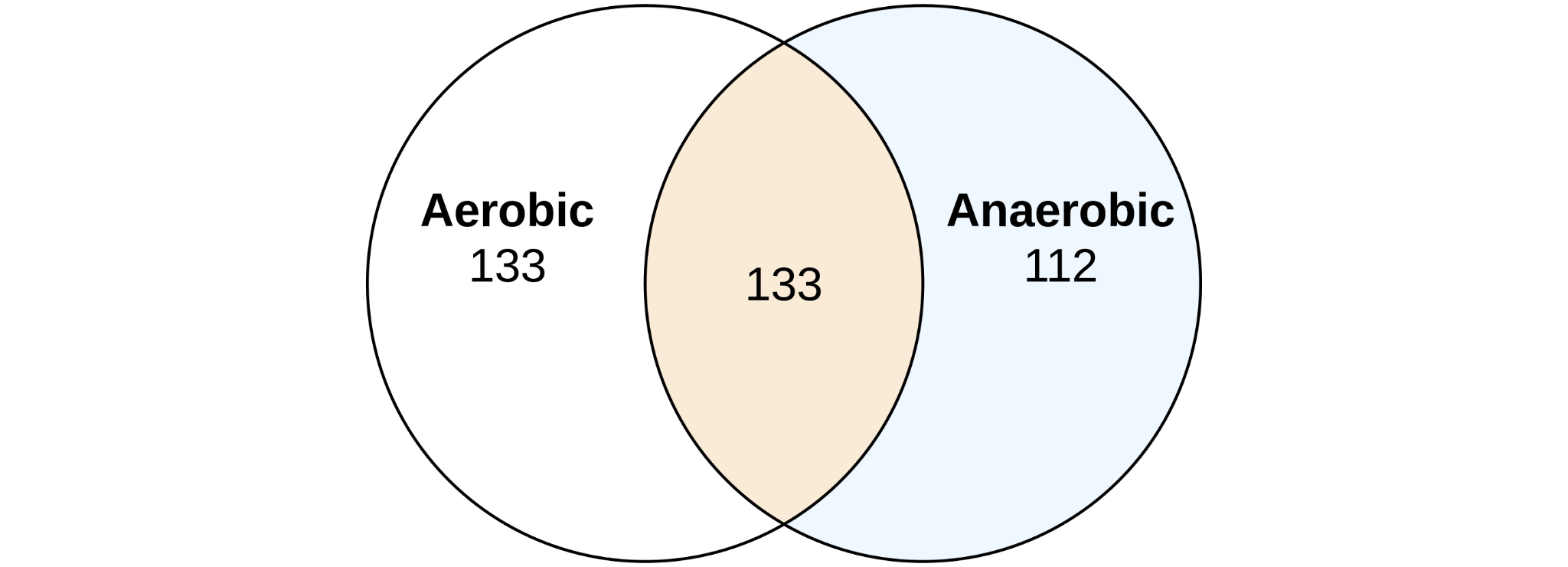


**Supplementary Figure 2.** Taxonomic composition of aerobic and anaerobic microbial consortia. Stacked bar plots displaying the relative abundance of bacterial families across aerobic (left) and anaerobic (right) samples. Each bar represents an individual sample, and colors indicate different microbial families as listed in the legend.


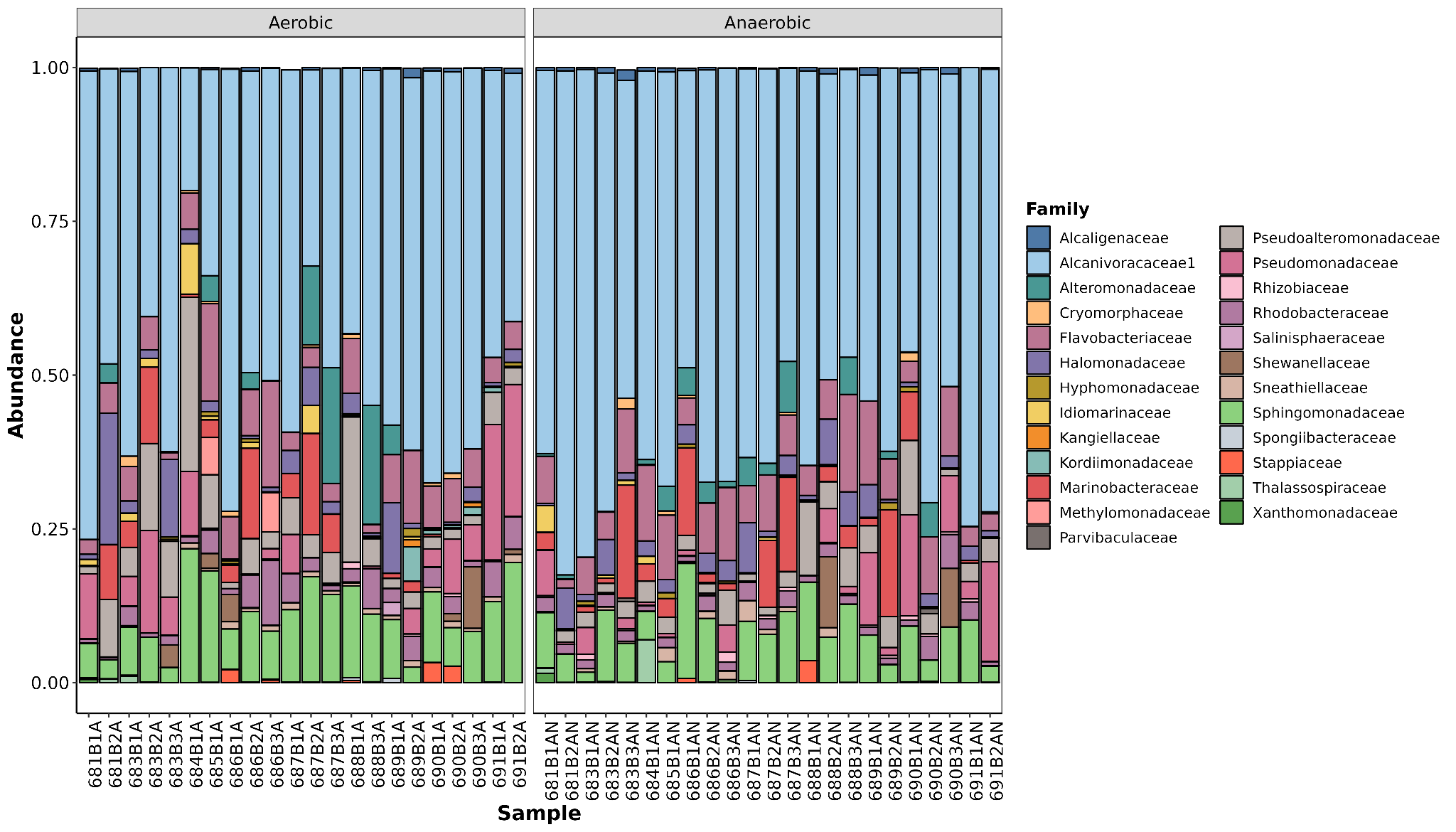


**Supplementary Figure 3.** Microbial community composition across sediment types and depths. Bar plots showing the relative abundance of microbial phyla across different sediment layers and geological features: Carbonate-rich sediment, Alpha Crucis Carbonate Ridge, and Pockmark fields. Each bar represents a specific layer: Superficial (S: 0–5 cm), Middle (M: 5–10 cm), Deep (D: 10–15 cm), and Subsurface (SB: 200–350 cm), with phyla colored according to the legend on the right.


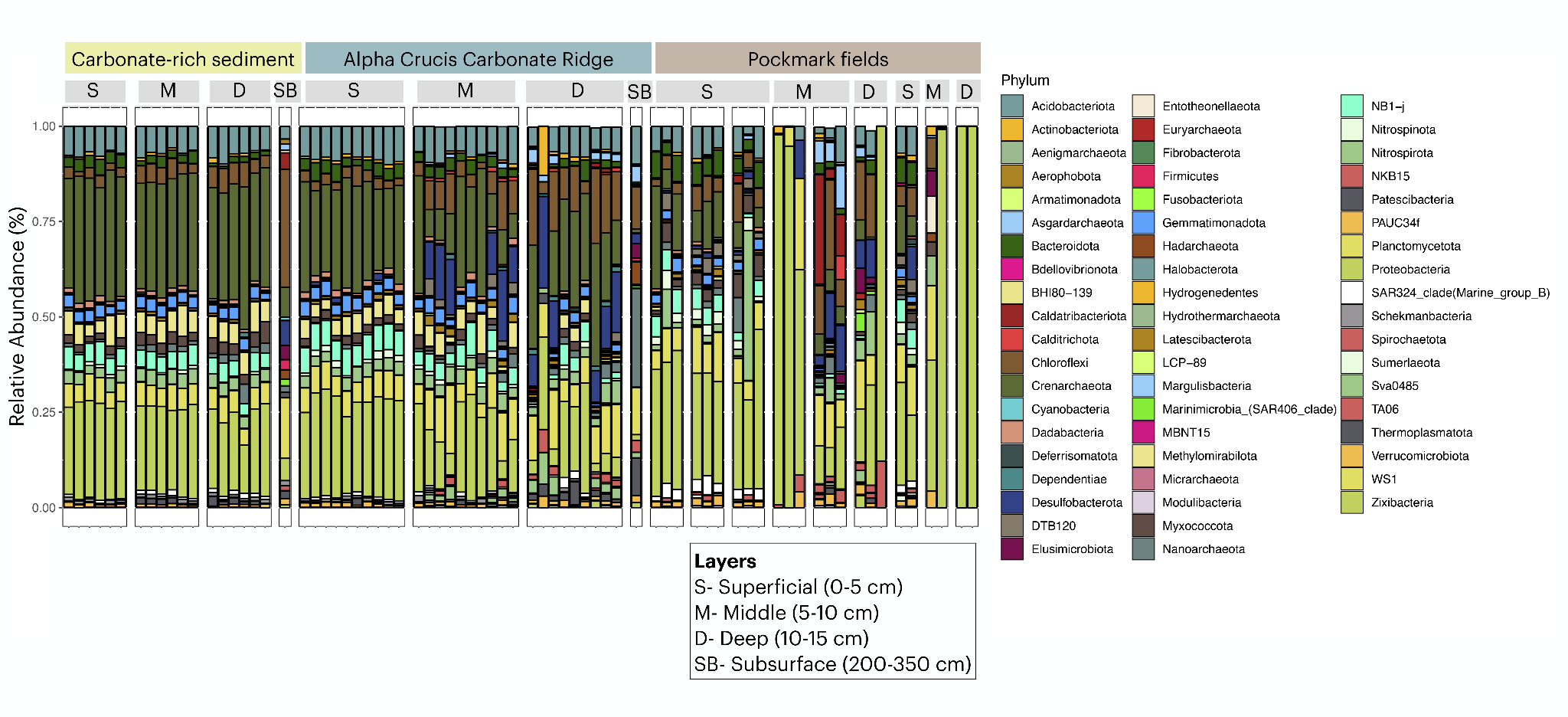


**Supplementary Table 3.** Summary of read counts before and after quality filtering in metagenomic sequencing. Total raw reads generated per sample and corresponding high-quality reads retained following quality control (QC) processing. All samples retained >98.9% of reads post-filtering, indicating robust data quality. Sample IDs reflect experimental designations.

| **Samples** | **N of total reads** | **Nº of reads after filtering** |
| --- | --- | --- |
| 685B1A | 65017782 | 64297341 |
| 686B3A | 74157411 | 73376395 |
| 684B1AN | 65894265 | 65079769 |
| 688B3AN | 74383372 | 73515119 |
| 683B3A | 76087279 | 75106427 |

**Supplementary Table 4.** Assembly statistics of metagenomic contigs generated with metaSPAdes. Summary of contig characteristics (≥500 bp) after *de novo* assembly, including: total contig count, cumulative assembly length (bp), maximum contig length (bp), GC content (%), N50 (contig length at which 50% of the assembly is contained in longer contigs), and L50 (number of contigs reaching N50).

| **Samples** | **Number of contigs** | **Full size**  **(bp)** | **Bigger contig (bp)** | **GC content**  **(%)** | **N50** | **L50** |
| --- | --- | --- | --- | --- | --- | --- |
| 685B1A | 5595 | 105525954 | 810464 | 51.63 | 58167 | 408 |
| 686B3A | 3525 | 79133215 | 1120593 | 56.36 | 96857 | 172 |
| 684B1AN | 6159 | 87859964 | 765301 | 56.65 | 46552 | 346 |
| 688B3AN | 4708 | 91733532 | 1885074 | 54.97 | 97869 | 190 |
| 683B3A | 2053 | 61374229 | 1011355 | 56.34 | 151495 | 102 |

**Supplementary Figure 4.** Taxonomic distribution of Metagenome-Assembled Genomes (MAGs) at the phylum and family levels. Bar plot showing the abundance of MAGs classified into major bacterial families. Colors represent dominant phyla: Proteobacteria (turquoise), Actinobacteriota (lilac), and Bacteroidota (pink). Families are ordered by MAG count (x-axis), with Sphingomonadaceae being the most abundant. The category "Unknown" denotes MAGs unclassified at the family level. "Koll-22" indicates an unresolved taxonomic group.


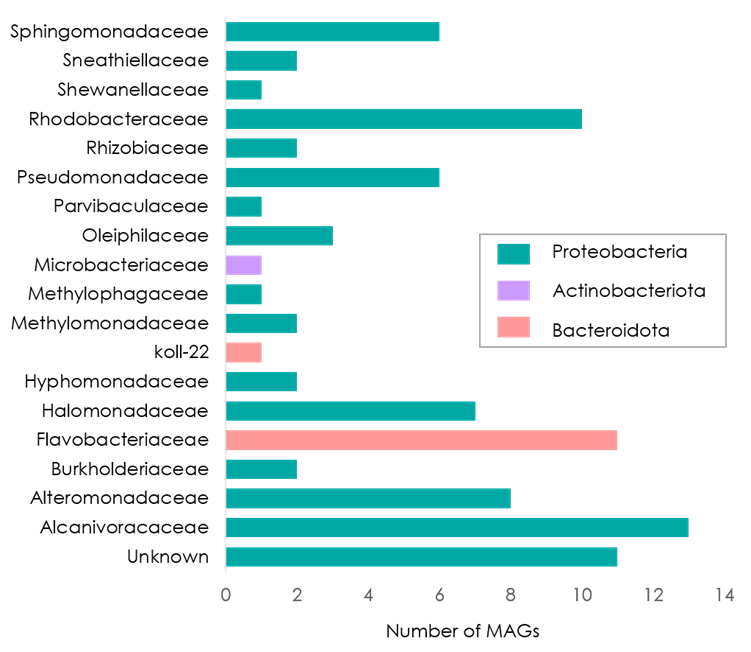


**Supplementary Table 5.**  Protein clustering statistics for Metagenome-Assembled Genomes (MAGs) and reference *Methylotuvimicrobium* genomes, showing total protein counts, clustered proteins (orthologous groups), and unclustered singleton proteins. MAGs LECOM 001 (5,109 proteins) and LECOM 002 (5,084 proteins) exhibit substantially higher protein recovery and clustering efficiency (≥92% clustered proteins; ≤1% singletons) compared to reference genomes, which show greater fragmentation (e.g., 265 singletons in *Methylotuvimicrobium* sp. wino1). The enhanced completeness of MAGs highlights successful genome reconstruction from metagenomic data.

| **Genome** | **Proteins** | **Clusters** | **Singletons** |
| --- | --- | --- | --- |
| *Methylotuvimicrobium* *crucis* LECOM 001 | 5109 | 4716 | 47 |
| *Methylotuvimicrobium* *crucis* LECOM 002 | 5084 | 4705 | 48 |
| *Methylotuvimicrobium* sp. wino1 | 4189 | 3615 | 265 |
| *Methylotuvimicrobium buryatense* strain 5GB1C | 4152 | 3546 | 195 |
| *Methylotuvimicrobium alcaliphilum* str. 20Z | 4086 | 3569 | 269 |
